# UA-Zero as a Uranyl Acetate Replacement When Diagnosing Primary Ciliary Dyskinesia by Transmission Electron Microscopy

**DOI:** 10.3390/diagnostics11061063

**Published:** 2021-06-09

**Authors:** Andreia Lucia Pinto, Ranjit Kaur Rai, Amelia Shoemark, Claire Hogg, Thomas Burgoyne

**Affiliations:** 1 Royal Brompton Hospital, Guy’s and St Thomas’ NHS Foundation Trust, London SW3 6NP, UK; A.Pinto@rbht.nhs.uk (A.L.P.); r.rai@rbht.nhs.uk (R.K.R.); a.shoemark@dundee.ac.uk (A.S.); c.hogg@rbht.nhs.uk (C.H.); 2 Department of Life Sciences, NOVA School of Science and Technology, 2829-516 Caparica, Portugal; 3 School of Medicine, University of Dundee, Dundee DD1 9SY, UK; 4 Department of Paediatrics, Imperial College London, London SW3 6LY, UK; 5 UCL Institute of Ophthalmology, University College London, London EC1V 9EL, UK

**Keywords:** primary ciliary dyskinesia, uranyl acetate, diagnosis, electron microscopy

## Abstract

Primary ciliary dyskinesia (PCD) is a disorder affecting motile cilia. An early accurate diagnosis helps prevent lung damage and preserve lung function. To make a diagnostic assessment, one of the commonly used methods that allows for the examination of ciliary ultrastructure is transmission electron microscopy (TEM). This allows for a quantitative assessment of ciliary components to identify defects associated with PCD. Heavy metal staining is required to provide a contrast when imaging cilia in the TEM. One of the most commonly used stains is uranyl acetate (UA). UA can be applied to cellular material before embedding (en bloc), or to ultrathin sections of embedded samples (grid staining). UA is radioactive and, due to growing safety concerns and restrictions by government bodies, universities and hospitals, it is essential to find a suitable alternative. We show UA-zero (UAZ), when used en bloc, provides a high contrast and is a suitable replacement for UA. PCD diagnostic experts, having reviewed ciliary cross-sections stained with UAZ en bloc, are confident that the staining and PCD defects are readily detectable similar to samples that have been stained with UA.

## 1. Introduction

**Figure 1:**
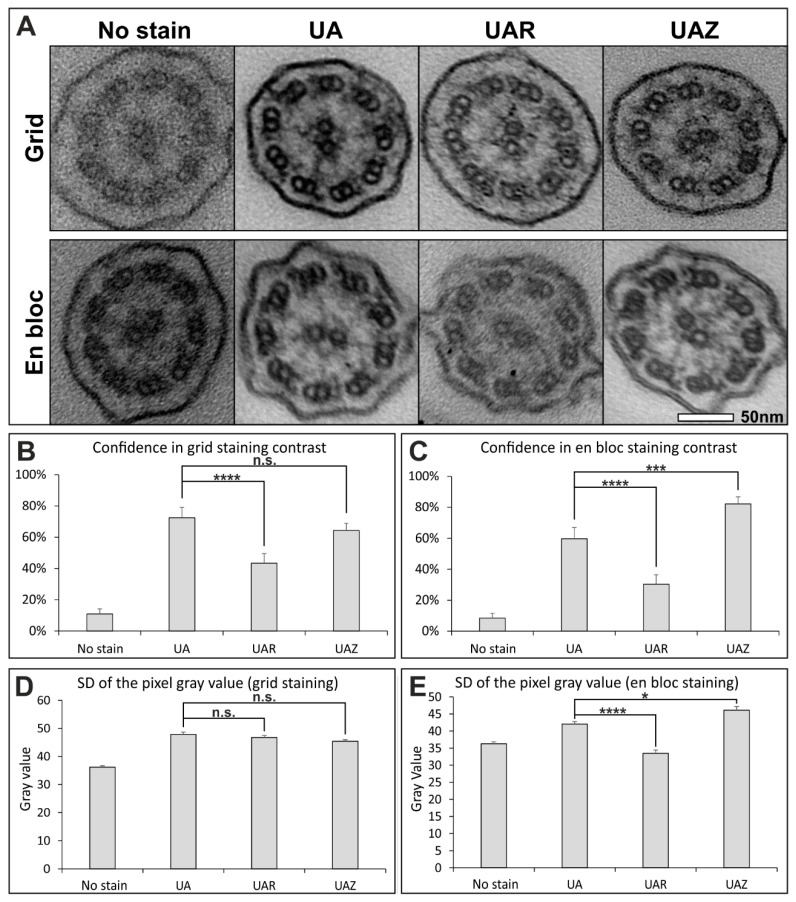
UAZ provides a good alternative to UA when examining ciliary cross-sections. (**A**) Images of ciliary cross-sections from samples prepared with grid staining or en bloc using one of following stains: lead citrate (LC) only, UA, UAR or UAZ. (**B**,**C**) Anonymised images were compared by 10 electron microscopy specialists and scored based on the confidence they had in the staining. (**B**) When staining sections on TEM grids, UA and UAZ gave the greater confidence in staining compared to UAR. No significant difference in confidence in staining was observed between UA and UAZ. (**C**) UAZ en bloc provided staining with the highest confidence results. (**D**,**E**) Standard deviation (SD) of the pixel gray value within images of ciliary cross-sections was determined to examine the contrast range. (**D**) No difference in the SD of the pixel gray value was observed between UA, UAR and UAZ. (**E**) There was a significantly greater SD of pixel gray value when comparing UAZ to UA en bloc staining. A lower standard deviation of the pixel gray value was found for UAR when compared to UA en bloc staining. (**B**–**E**) Standard error of mean (SEM) shown and statistical significance was determined using Student’s *t*-test (n.s. *p* > 0.05 * *p* < 0.05 *** *p* < 0.01 **** *p* < 0.0001).

Primary Ciliary Dyskinesia (PCD) is a genetic disease affecting motile cilia. Symptoms include chronic lung disease, rhinosinusitis, hearing impairment and subfertility. Diagnosis of PCD is confirmed by the identification of a ciliary ultrastructural defect by transmission electron microscopy (TEM) or identification of bi-allelic pathogenic mutations in a known PCD gene [1,2,3] in combination with other diagnostic tests (high-speed video analysis, immunofluorescence labelling and nasal nitric oxide measurements). The normal ultrastructure of respiratory cilia is shown in Figure 1A. The hallmark defects used to diagnose PCD include outer dynein arm defects, and outer and inner dynein arm defects, inner dynein arm defects with microtubular disorganisation and central complex defects. The dynein arms are small structures of approximately 10 nm in length [4] and, in order to visualise these structures accurately at the resolution of diagnostic TEM, good contrast (high signal to noise ratio) is essential. Contrast is usually achieved using a combination of heavy metal stains including osmium tetroxide, lead citrate, and uranyl acetate (UA). 

UA is derived from depleted uranium and is radioactive (alpha emitter). It is extensively used as a TEM stain, either en bloc during sample preparation or staining ultrathin sections on electron microscopy grids [5]. Due to increasing concerns regarding the use of radioactive material for personal safety and difficulties disposing UA waste, many institutes have strict regulations on its use (e.g., United Kingdom Government Ionising Radiation Regulations 2017). Justification for the procurement and use of UA for diagnostic TEM has become increasingly difficult, and there is a global drive to find a safer, non-radioactive replacement. Alternative reagents, including platinum-blue [6], oolong tea extract [7] and hafnium chloride [8], have been trialed as alternative stains for TEM, but are generally inferior to UA and their use is not widespread. More recently, commercially available regents have become available, including UA-Zero (UAZ, Agar Scientific Ltd, Stansted, Essex, UK) and UAR-EMS (UAR, Electron Microscopy Sciences, Hatfield, PA, USA). These are pre-mixed solutions which contain rare earth elements (lanthanides) for contrast staining. UAZ includes ytterbium(III) chloride hexahydrate, and UAR has both samarium triacetate and gadolinium triacetate [9]. It is important to validate any changes to diagnostic procedures to ensure that providing a safer contrast stain does not compromise the quality of diagnostic ultrastructural studies. Here, we compare two UA replacement stains to UA to determine their suitability in the diagnosis of PCD by TEM. 

## 2. Materials and Methods

### 2.1. Ethical Approval

The study was approved by the ethics review board of the Institute of Child Health/Great Ormond Street Hospital, London (UK) (08/H0713/82 15/10/2008). All subjects gave informed signed consent for genetic testing and the use of surplus diagnostic samples for the improvement and development of new diagnostic methods.

### 2.2. Preparation of Nasal Brushing Biopsies for Transmission Electron Microscopy

3 mm Cytology Brushes (ConMed, Largo, FL, USA) were used to take biopsies of the respiratory epithelium from the inferior nasal turbinate of four patients with genetically confirmed PCD (see Table 1 for mutations), patients referred for diagnostic testing and a healthy control. The four patients were referred for diagnostic testing and a diagnosis of PCD was confirm through the diagnostic pathway at the Royal Brompton Hospital. This included assessment by high-speed video microscopy to assess ciliary beat pattern and frequency and measurement of nasal nitric oxide, as well as electron microscopy and genetic testing. All four patients have mutations and ultrastructural defects that were previously described [10,11].

Nasal brushings were fixed with 2.5% glutaraldehyde (VWR International, Lutterworth, Leicestershire, UK) in 0.05 M sodium cacodylate buffer (Agar Scientific, Stansted, Essex, UK) at a pH 7.4 and stored at 4 °C overnight or for up to 2 weeks. Samples were washed in 0.05 M sodium cacodylate buffer and embedded in 1% agar (Oxoid, Basingstoke, Hampshire, UK) before incubation in 1% aqueous osmium tetroxide (TAAB Laboratory Equipment, Aldermaston, Berkshire, UK) for 1 h, followed by washes in distilled water. At this point, the healthy control nasal brushing biopsy was split up into four separate samples. The specimens were prepared using one of the following en bloc stains: (i) UA, (ii) UAZ, (iii) UAR, or (iv) no stain. The details of each of these stains is provided in Table 2. All samples were dehydrated in increasing concentrations of ethanol (50%, 70%, 90%, 100%), followed by propylene oxide and a mixture of propylene oxide and resin (1:1), before infiltration and embedding in araldite resin (Agar Scientific, Stansted, Essex, UK). Polymerization was performed at 60 °C for 48 h, and ultrathin sections of 80 nm were obtained using a Reichert Ultracut E ultramicrotome and collected on copper mesh grids (Agar Scientific, Stansted, Essex, UK). For the samples prepared with no en bloc staining, sections were collected onto grids and stained with either; (i) UA (Agar Scientific, Stansted, Essex, UK), (ii) UAZ (Agar Scientific, Stansted, Essex, UK), (iii) UAR (Electron Microscopy Sciences, Hatfield, PA, USA) or (iv) left unstained. All except the unstained were counter-stained with Reynold’s lead citrate (Agar Scientific, Stansted, Essex, UK), and the details of the methods used for each of these stains are provided in Table 3. Some of the sections collected from the UAZ en bloc were further grid-stained with UAZ. Images were acquired using a JEOL 1400 plus TEM (JEOL ltd, Tokyo, Japan) at an accelerating voltage of 120 kV. Digital images were obtained using an Advanced Microscopy Technologies (AMT) XR16 bottom mid-mount digital camera (AMT Imaging Direct, Woburn, MA, USA). The camera software has an autogain system that remaps the pixels values so that they extend across a larger range, making it easier to differentiate between lighter and darker pixels. When acquiring images, a fast Fourier transform was generated to make sure images were all taken 0.7 μm from defocus, and that there was no drift or beam astigmatism (as shown in Supplementary Materials Figure S1).

### 2.3. Analysis of Diagnostic Results

Diagnostic results from the assessment of nasal biopsy samples prepared for TEM with either en bloc UAZ (*N* = 58) or UA (*N* = 58) grid stain were examined. These were all patients referred to the Royal Brompton Hospital for diagnostic testing, including those that were given a positive and negative diagnosis for PCD. The diagnostic assessment by electron microscopy included counting: cilia with a normal (9 + 2), abnormal microtubule arrangement (8 + 2, 8 + 1 and 7 + 2), extra microtubules, missing central pair, compound cilia and dynein arm defect. The diagnostic results were analysed using Microsoft Excel.

### 2.4. Image Analysis

Cilia were cropped out of the images acquired from samples prepared with the different en bloc and grid stains using Cilia Crop, part of the PCD Detect toolkit [10]. The cropped images were anonymised using an in-house program designed using MATLAB to allocate a random number for file names (doi:10.5522/04/14369258).

A total of 20 images per staining condition were analysed by 10 electron microscopists experienced in PCD diagnosis and blinded to which stain was used. Images were ranked as ‘good’, ‘useable’ or ‘unusable’ for PCD diagnosis. These terms were converted into the corresponding scores; 100%, 50% and 0%, respectively. This allowed for assessment of the confidence the specialists had in the different grid or en bloc stains. 

To analyse the contrast range of images of cilia from the different staining methods the standard deviation of the pixel gray value was calculated using ImageJ (NIH). All images are 8-bit greyscale, where 0 is black and 255 is white. Statistical significance was determined for the survey results and standard deviation of the pixel gray value using Student’s *t*-tests. 

## 3. Results

The results of a survey comparing the different staining methods to determine their suitability for diagnosing PCD is shown in Figure 1. Representative images of the different methods are shown in Figure 1A and Supplementary Materials Figure S2, and it can be clearly seen that all staining methods give better contrast than no stain. The assessment of ciliary cross-sections by 10 electron microscopists who routinely analyse cilia cross-sections to diagnose PCD revealed similar confidence when comparing UAZ to UA grid stain but lower confidence in UAR (Figure 1B). UAZ en bloc provided significantly higher confidence than both en bloc UA and UAR of over 80% (Figure 1C). The standard deviation (SD) of the pixel gray value was calculated from images of the ciliary cross-sections (Figure 1D,E). All three stains, when applied to sections, gave similar standard deviations of the pixel gray value (Figure 1D). UAZ en bloc staining gave a significantly higher SD of the pixel value compared to UA, indicating greater image contrast (Figure 1D), whereas UAR gave a significantly lower standard deviation of the pixel gray value compared to UA. 

**Figure 2:**
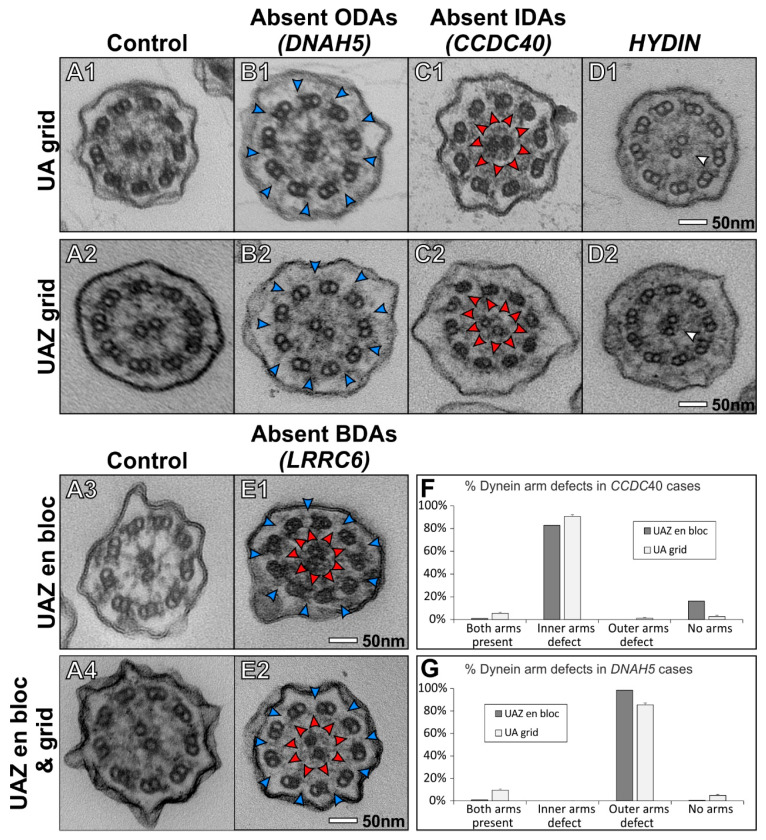
Key structural features for diagnosing PCD are visible when using UAZ to stain grids or en bloc. (**A**) The control cilia show a normal cilia ultrastructure that includes the presence of outer and inner dynein arms and a complete central pair complex. (**B**,**C**) Outer (blue arrowheads) or inner dynein arms (red arrowheads) are absent in patients that have disease-causing variants in *DNAH5* or *CCDC40* when staining with UA or UAZ on grid. (**D**) The loss of the C2b component (white arrowheads) of the central pair can be detected in an *HYDIN* case when staining grids with UAZ and UA. (**E**) The loss of both dynein arms (blue and red arrowheads) can be seen in an *LRRC6* case when staining with UAZ en bloc with and without an additional grid stain. The additional stain does not compromise image contrast. (**F**,**G**) Diagnostic results from a single UAZ en bloc prepared samples compared to UA grid-stained samples, showing a similar level of detection of dynein arm defects (SEM shown). (**F**) Diagnostic assessment of patients that have pathogenic variants in *CCDC40* (UAZ en bloc *N* = 1 and UA en bloc *N* = 5). (**G**) Diagnostic results from patients with pathogenic variants in *DNAH5* (UAZ en bloc *N* = 1 and UA en bloc *N* = 6).

The staining methods were further assessed by examining ciliary cross-sections from PCD patients that have known pathogenic variants causing an absence of ciliary structures, as described in Table 1. The missing axoneme components that include ODA, IDA and C2b component of the CP were detectable in patient samples when using UAZ grid staining (Figure 2A–D and Supplementary Materials Figure S3). UAZ en bloc staining, which provides better confidence in terms of staining quality than the use of UA (Figure 1C), provided a high contrast and allowed for the detection of inner and outer dynein arm defects (Figure 2A3,E1 and Supplementary Materials Figure S3). The addition of UAZ grid staining on samples that had been prepared with UAZ en bloc did not improve the contrast further (Figure 2A4,E2 and Supplementary Materials Figure S3).

Following the results of our blinded survey, we integrated UAZ staining into our clinical practice. UAZ en bloc was performed on 58 patient samples and, out of these, 11 tested positive for PCD. We audited our clinical practice, where up to 100 cilia are counted per case, to make a diagnosis. Out of the total cilia cross-section examined per case, 52.60 ± 1.82% with UA and 53.48 ± 1.54% with UAZ (shown as mean ± standard error of the mean) could be assessed for dynein arm defects. This indicates that the UAZ stain change in protocol did not impact the quality of our clinical practice. Furthermore, two cases had been previously assessed with UA and were reassessed with UAZ staining. These showed gene mutations in DNAH5 and CCDC40. Quantification of these samples showed a similar detection of dynein arms defects to previous cases that were UA grid-stained (without en bloc staining), as shown in Figure 2F,G.

**Figure 3:**
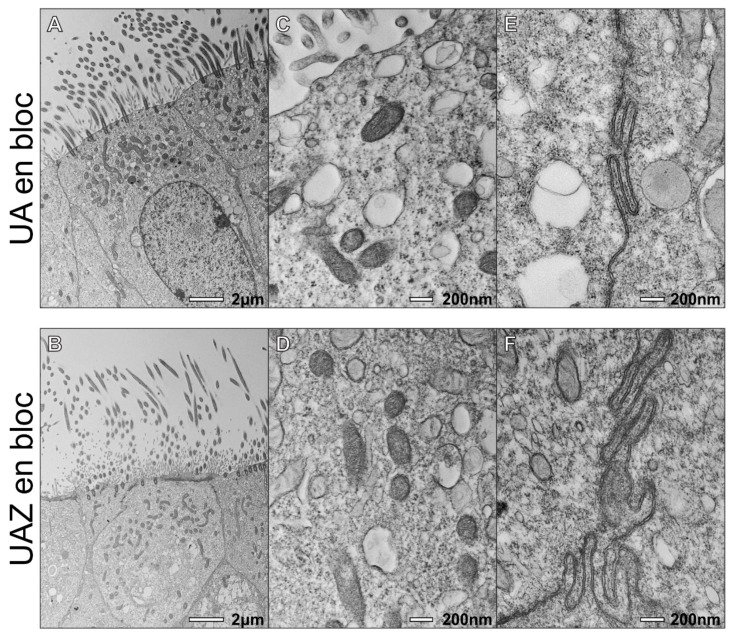
UAZ en bloc provides sample staining comparable to UA en bloc. Images of respiratory epithelium prepared from a nasal brushing with (**A**–**C**) UA en bloc and (**D**–**F**) UAZ en bloc. (**A**,**D**) Low magnification view of ciliated respiratory epithelium shows a similar level of contrast between the two stains. (**B**,**E**) There is clear staining of intercellular compartments and organelles including mitochondria. (**C**–**F**) Both stains provide good membrane staining, as demonstrated by the plasma membrane staining at junctions between cells.

In addition to ciliary ultrastructure, it is important for PCD diagnosis to assess the health of the cells and quality of the sample; therefore, we assessed other cellular structures in the samples. UAZ en bloc provides good contrast staining of other organelles and cellular membrane, comparable to the UA en bloc (Figure 3). At a lower magnification, the contents of the cell can be clearly seen with both UAZ and UA en bloc staining (Figure 3A,D). At higher magnification, organelles such as mitochondria have good contrast, and membrane at cellular junctions are clear and of similar quality for both stains (Figure 3C–F).

## 4. Discussion

We show that en bloc staining with UAZ provides a promising replacement for UA for the assessment of ciliary ultrastructure. It provides a comparable contrast to UA and allows for imaging of the key ciliary components that are linked to PCD.

UAZ en bloc staining applied during sample preparation resulted in TEM images with a high signal to noise ratio that, when assessed by PCD diagnostic specialists, gave more confidence in terms of staining and ability to detect ciliary features compared to UA. When using UAZ en bloc staining for a PCD patient with a pathogenic variant in *LRRC6*, which encodes a protein required for the transport of dynein arms [12], the absence of the IDAs and ODAs was clear. Patients that have been diagnosed with ODA or/and IDA defects associated with pathogenic variant in *DNAH5* or *CCDC40* show the detection of a similar ratio of defects to similar cases that were prepared with UA grid staining.

UAZ grid staining conferred similar confidence to UA and was found to be suitable for diagnosing PCD, as absent ciliary structures were detectable in patient samples. This includes cilia from patients with pathogenic variants in *DNAH5* associated with a loss of ODAs [13], *CCDC40* which leads to loss of IDAs and microtubular disarrangement [14] and *HYDIN* which causes the loss of the C2b component of the central pair [15]. Based on our findings, we suggest UAZ could be used en bloc as a UA replacement in diagnosing PCD, and UAZ grid staining can be used when samples have already been embedded and UA is unavailable. 

This is the first time that UA replacement stains have been compared for the diagnosis of PCD. This was done blinded and included PCD samples from genotyped patients. The assessment was done by several experts from different centres that specialise in the diagnosis of PCD. We have prepared 58 diagnostic samples with en bloc staining and the assessment of absent ciliary structures and other defects reflects the results found from our previous UA grid staining protocol.

Only a single control nasal brushing sample was assessed in the survey that was split up and prepared using different staining methods. As centres use a range of sampling techniques and different methodology for processing and assessment by TEM, UAZ en bloc would need to be validated locally before implementation in other centres. 

We predict that the sensitivity and specificity of TEM in the diagnosis of PCD could improve when using UAZ compared to more traditional UA methods, due to the greater confidence we found in the staining of cilia. Due to the safety concerns and difficulties in purchasing, handling and storing of UA, this makes UAZ a suitable replacement regent for assisting in the diagnosis of PCD. 

## 5. Conclusions

For the first time, we compare different staining methods for the diagnosis of PCD by electron microscopy. UAZ provides a promising and safer alternative to UA for the assessment of ciliary ultrastructure. We show that UAZ allows for the detection of ciliary structural defects associated with PCD and, when used in the diagnostic pathway, has similar results to UA staining.

## Figures and Tables

**Table 1:** Genetic information of PCD subjects that had TEM samples stained in this study.

PCD Gene	Patient Symptoms	Axoneme Structure Affected	Genotype
DNAH5	Persistent rhinitis and prolonged otorrhea post grommet insertion	Defect of ODAs	Unsolved—Heterozygous DNAH5 NM_001369.2: c.7477G > C (p.Glu2493Arg)
CCDC40	-	IDAs and microtubular disorganisation	CCDC40 NM_017950.3: c.2712–1G > T + c.2712–1G > T
HYDIN	Normal situs, chronic wet cough, rhinitis, and otorrhea	CP C2b component	HYDIN NM_001270974.1: c.8487del (p.Pro2830Hisfs*23) + c.8489C > A (p.Pro2830Gln)
LRRC6	Chronic wet cough and affected family members	Defect of both ODAs and IDAs	LRRC6 NM_012472.3: c630del (p.Trp210Cysfs*12) + c630del (p.Trp210Cysfs*12)

Translation termination codon is referred to by *.

**Table 2:** Methods of en bloc staining used to prepare nasal brushing for assessment of ciliary ultrastructure by TEM.

En Bloc Stain	Method
No stain	No stain added during sample preparation
UA	1% aqueous UA (Agar Scientific Ltd.) for 30 min
UAR	UAR (Electron Microscopy Sciences) diluted 1:4 in distilled water and applied for 30 min
UAZ	undiluted UAZ (Agar Scientific) for 30 min

**Table 3:** Methods of grid staining used to prepare nasal brushing for assessment of ciliary ultrastructure by TEM.

Grid Stain	Method
No stain	No stain
UA	2% aqueous UA for 7 min and Reynold’s lead citrate for 10 min
UAR	UAR (Electron Microscopy Sciences) diluted 1:4 in distilled water and applied for 30 min and Reynold’s lead citrate Reynold’s lead citrate
UAZ	undiluted UAZ for 7 min and Reynold’s lead citrate for 10 min

## Data Availability

No new datasets were created or analyzed in this study. Data sharing is not applicable to this article. The software used to randomize images names is publicly available and can be found here [doi:10.5522/04/14369258].

## Supplementary Materials

The following are available online at https://www.mdpi.com/article/10.3390/diagnostics11061063/s1, Figure S1: Representative fast Fourier transform (FFT) used to set the defocus of images acquired for the survey, Figure S2: Further example of electron microscopy images of cilia from samples that have been prepared with different stains, Figure S3: Further example of electron microscopy images of cilia from PCD patients.
