# Supplementary material for: UA-Zero as a Uranyl Acetate Replacement When Diagnosing Primary Ciliary Dyskinesia by Transmission Electron Microscopy
Source: Diagnostics (Basel). 2021 Jun 9;11(6):1063. doi: 10.3390/diagnostics11061063 (PMC8229773; doi:10.3390/diagnostics11061063)
Supplement: Supplementary file 1 [file diagnostics-11-01063-s001.zip › Figure S3.pdf]

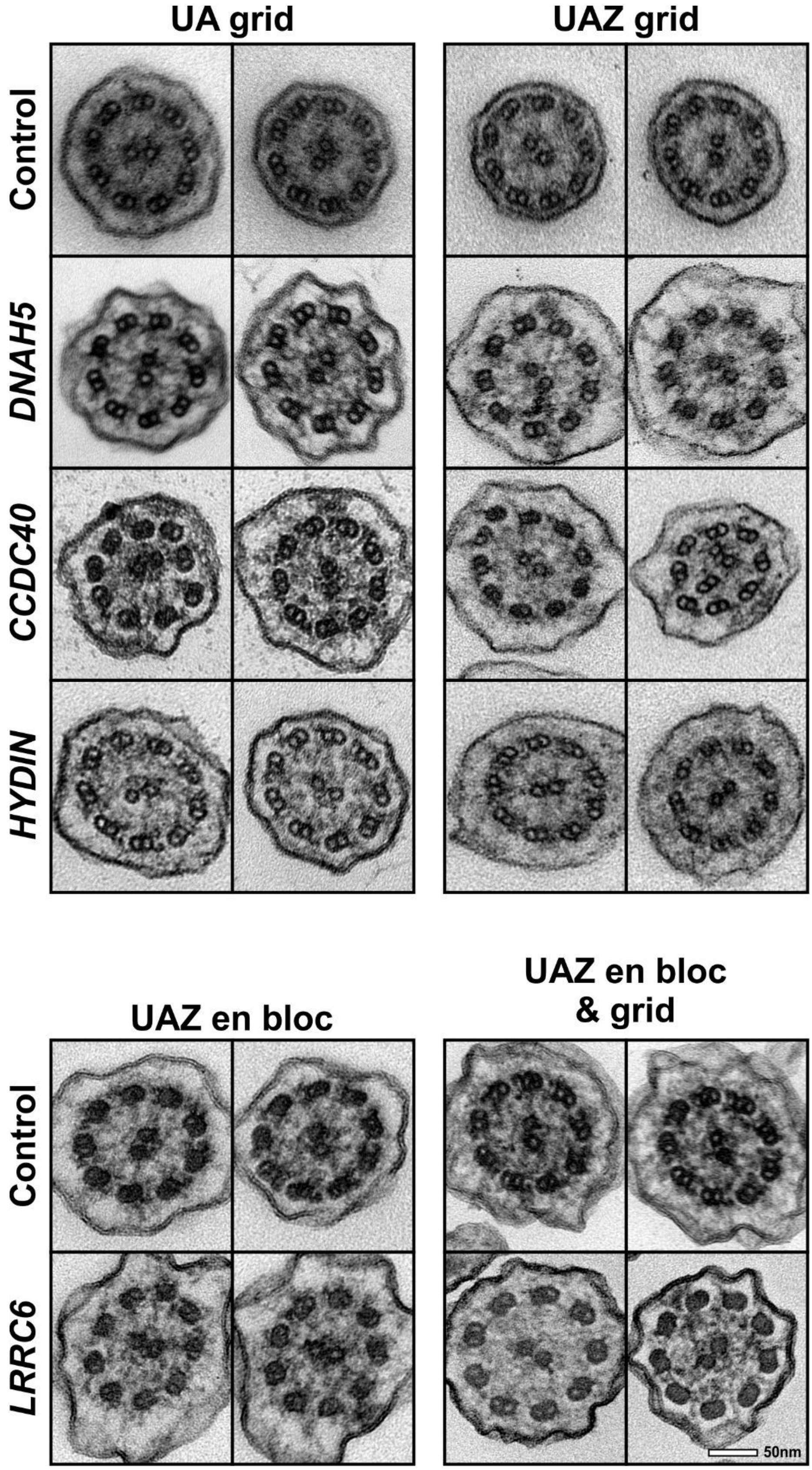

Supplementary Figure S3. Further example of electron microscopy images of cilia from PCD patients. Patients include pathogenic mutations in *DNAH5, CCDC40, HYDIN and LRRC6* that have defects in the outer dynein arms, inner dynein arms, central pair complex and both dynein respectively. Samples were stained en bloc during sample preparation with UA or UAZ and/or on ultrathin sections on grids.
