# Supplementary material for: UA-Zero as a Uranyl Acetate Replacement When Diagnosing Primary Ciliary Dyskinesia by Transmission Electron Microscopy
Source: Diagnostics (Basel). 2021 Jun 9;11(6):1063. doi: 10.3390/diagnostics11061063 (PMC8229773; doi:10.3390/diagnostics11061063)
Supplement: Supplementary file 1 [file diagnostics-11-01063-s001.zip › Figure S1.pdf]

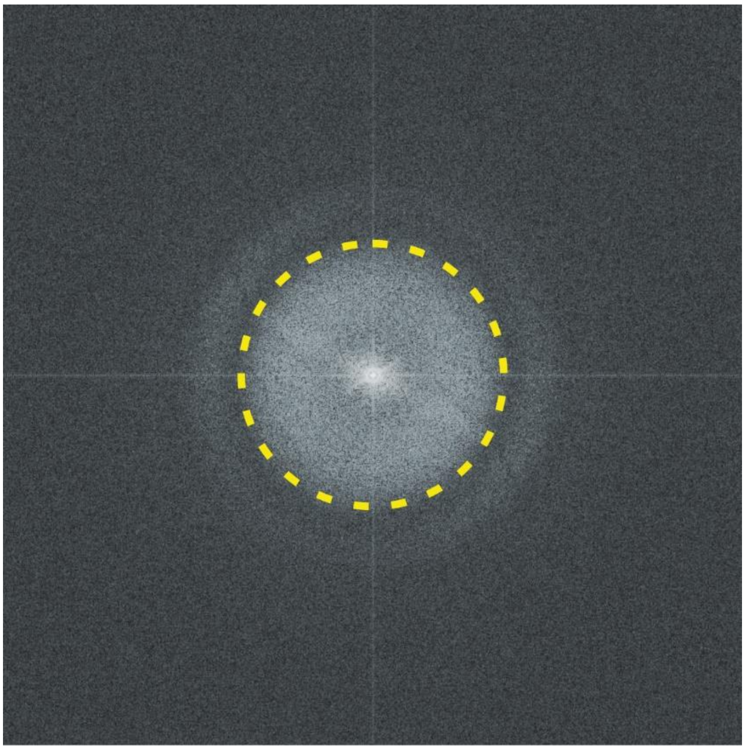

Supplementary Figure S1. Representative fast Fourier transform (FFT) used to set the defocus of images acquired for the survey. All images were acquired with a defocus of 0.7μm avoiding beam astigmatism and sample drift.
