# Supplementary material for: UA-Zero as a Uranyl Acetate Replacement When Diagnosing Primary Ciliary Dyskinesia by Transmission Electron Microscopy
Source: Diagnostics (Basel). 2021 Jun 9;11(6):1063. doi: 10.3390/diagnostics11061063 (PMC8229773; doi:10.3390/diagnostics11061063)
Supplement: Supplementary file 1 [file diagnostics-11-01063-s001.zip › Figure S2.pdf]

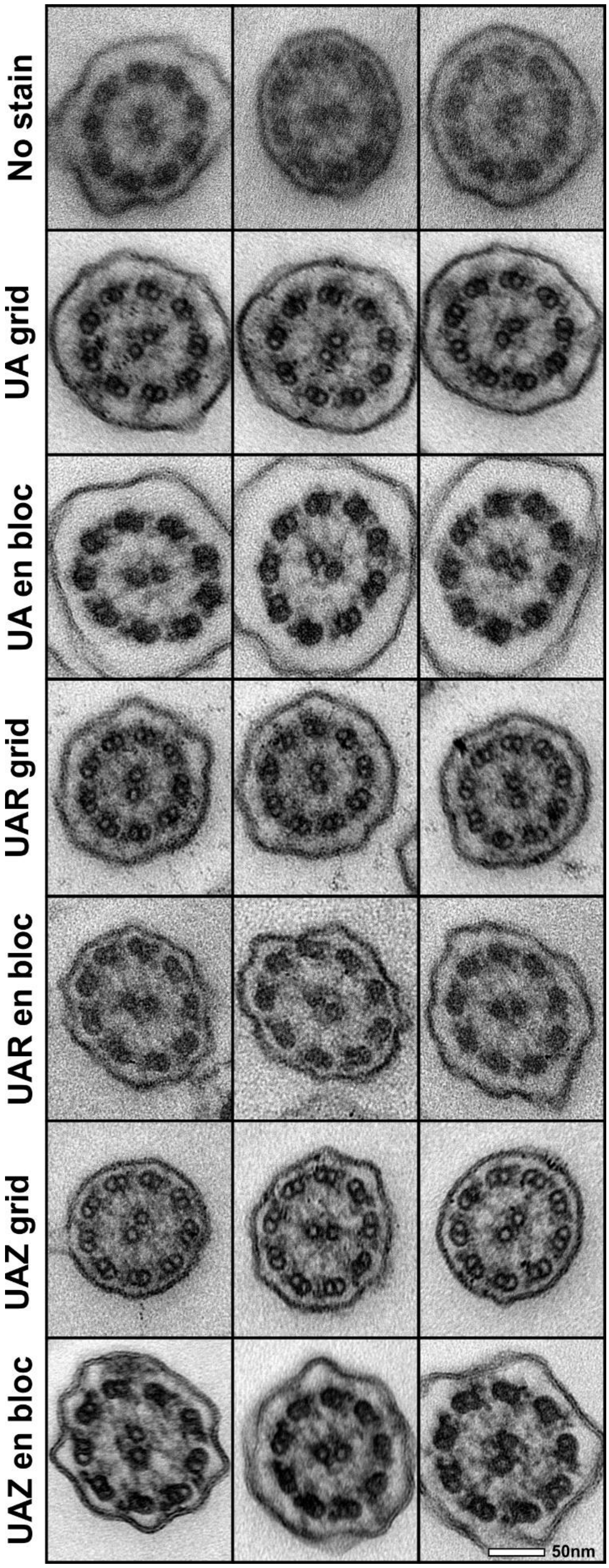

Supplementary Figure S2. Further example of electron microscopy images of cilia from samples that have been prepared with different stains. UA, UAZ or UAR were applied either during sample preparation (en bloc) or to stain ultrathin sections on grids.
